# Targeting CXCL12/CXCR4 and myeloid cells to improve the therapeutic ratio in patient-derived cervical cancer models treated with radio-chemotherapy

**DOI:** 10.1038/s41416-019-0497-3

**Published:** 2019-06-26

**Authors:** Magali Lecavalier-Barsoum, Naz Chaudary, Kathy Han, Melania Pintilie, Richard P. Hill, Michael Milosevic

**Affiliations:** 1 Segal Cancer Centre, Jewish General Hospital and McGill University, Montreal, Canada; 2 University Health Network and Princess Margaret Cancer Centre, Toronto, Canada; 3 Department of Radiation Oncology, University of Toronto, Toronto, Canada; 4 Institute of Medical Science, University of Toronto, Toronto, Canada; 5 Dalla Lana School of Public Health, University of Toronto, Toronto, Canada; 6 Department of Medical Biophysics, University of Toronto, Toronto, Canada

**Keywords:** Radiotherapy, Cancer microenvironment, Tumour immunology

## Abstract

**Background:**

The CXCL12/CXCR4 chemokine pathway is involved in cervical cancer pathogenesis and radiation treatment (RT) response. We previously reported that radiochemotherapy (RTCT) and concurrent administration of the CXCR4 inhibitor plerixafor improved primary tumour response. The aims of this study were to determine optimal sequencing of RTCT and plerixafor, the mechanisms responsible for improved response and the effect of plerixafor on late intestinal toxicity.

**Methods:**

Orthotopic cervical cancer xenografts were treated with RTCT (30 Gy in 2 Gy fractions and cisplatin) with or without concurrent, adjuvant or continuous plerixafor. The endpoints were growth delay and molecular and immune cell changes at the end of treatment. Late intestinal toxicity was assessed by histologic examination of the rectum 90 days after a single 20 Gy fraction.

**Results:**

RTCT increased CXCL12/CXCR4 signalling and the intratumoral accumulation of myeloid cells; the addition of plerixafor mitigated these effects. All of the RTCT and plerixafor arms showed prolonged tumour growth delay compared to RTCT alone, with the adjuvant arm showing the greatest improvement. Plerixafor also reduced late intestinal toxicity.

**Conclusion:**

Adding Plerixafor to RTCT blunts treatment-induced increases in CXCL12/CXCR4 signalling, improves primary tumour response and reduces intestinal side effects. This combination warrants testing in future clinical trials.

## Background

Cervical cancer is the fourth leading cause of cancer death in women worldwide despite improvements in cervical screening and human papilloma virus (HPV) vaccination over the past decades.^1^ Approximately one-half of cervical cancer cases are diagnosed at a locally advanced stage for which surgery is not recommended.^2^ However, even these patients are potentially curable with radiotherapy and concurrent platinum-based chemotherapy (RTCT). When tumours progress locally after RTCT or when metastases develop, treatment options are often limited and ineffective. There is an important need for new therapeutic approaches to overcome radiation treatment resistance, prevent metastases and further improve cure rates.

Recently, the CXCL12/CXCR4 pathway has emerged as being of particular interest and relevance in cervical cancer as outlined in a recent review.^3^ It has been implicated in HPV infection and cervical carcinogenesis, malignant progression, the development of metastases and RT response.^3^ Our group previously reported that concurrent treatment of cervical cancer patient-derived xenografts (PDXs) with RTCT and the CXCR4 inhibitor plerixafor (AMD3100) produced substantial tumour growth delay and reduced lymph node metastases without increasing early (acute) intestinal toxicity compared to RTCT alone.^4^ This report builds on these findings by investigating different ways of sequencing RTCT and plerixafor for optimal efficacy, and the mechanisms responsible for improved treatment response as a foundation for future phase I/II clinical trials. We also evaluated long-term tumour control with the combination of RTCT and plerixafor, and the effect of plerixafor on late intestinal toxicity, the most common serious side effect of RTCT after treatment of cervical cancer.

## Methods

### Mice

Six- to 8-week-old female NOD-Rag1nullIL2rgnull (NRG) female mice (impaired adaptive immunity but intact chemokine pathways and myeloid cell immunity) and C57BL/6 mice (fully immunocompetent)^5^ were bred, housed and treated in accordance with protocols that conform to the Canadian Council on Animal Care.

### Patient-derived, orthotopic cervical cancer xenografts (PDXs)

The two PDX models used for these experiments were developed from clinical cervical cancer biopsies at the Princess Margaret Cancer Centre and grown orthotopically in the cervixes of mice as previously described.^6,7^ The radiation treatment growth delay experiments were done using OCICx 20 to maintain continuity with our previous studies.^4^ This PDX has been extensively characterised by our group.^6–8^ The tumour eradication experiments were done using OCICx 3. This PDX was selected because it has a radiation dose-response characteristic (data not shown) that results in a small proportion of tumour ‘cures’ with RTCT alone (50 Gy + concurrent cisplatin), making it better suited for evaluating long-term disease eradication with the addition of plerixafor. In general, these PDX models have been shown to mirror the clinical and biological behaviour of cervical cancer in patients, including the development of lymph node metastases, and respond similarly to RTCT.^6–8^

### Radiation treatment and tumour growth delay

All of the imaging and RT experiments were performed using a dedicated 225 kVp small animal irradiator and integrated cone-beam CT imager (XRAD225, Precision X-Ray, Connecticut) with the mice anaesthetised and immobilised in a lucite jig. Following implantation, the size of the cervical PDXs was monitored weekly using CT imaging. Tumour-bearing mice were randomly assigned to control or experimental groups when the tumours reached a size of 5–7 mm.

CT-guided, fractionated RT regimens reflective of clinical practice were used for the tumour growth delay studies. RT was planned using pre-treatment CT images to identify the cervical tumour volume. Customised treatment plans were developed using circular collimators 8 mm in diameter and multiple beams with roughly equal angular distribution around the tumour isocenter. The RT dose was 30 or 50 Gy in 2 Gy fractions at a dose rate of 3 Gy/minute, delivered Monday to Friday for 3 or 5 weeks. Cisplatin 4 mg/kg was administered intraperitoneally (ip) one day each week during RT. Plerixafor (Epsilon-Chimie, France) 5 mg/kg/day was administered by continuous subcutaneous (sc) infusion using implanted osmotic pumps (ALZET, California) concurrently with RTCT for 3 or 5 weeks, adjuvantly (RTCT alone for 3 weeks followed by plerixafor alone for 3 or 6 weeks) or continuously (RTCT and plerixafor for 3 weeks followed by an additional 3 weeks of plerixafor alone). Combinations of cisplatin alone and plerixafor were not investigated. While previous reports have suggested that plerixafor may enhance the effectiveness of chemotherapy,^9,10^ cisplatin alone is not standard-of-care treatment for newly diagnosed patients with potentially curable cervical and was therefore beyond the scope of this study.

Mice were followed after treatment with serial CT imaging to monitor tumour regrowth and were euthanised when the tumours regrew to a size of 1–1.5 cm. The tumour volume was calculated from the largest diameter in each dimensional plane (*x*, *y*, *z*) based on an ellipsoid model of volume. Two observers independently estimated tumour size from the CT images. Disagreements were resolved by joint review of the images.

### End-of-treatment tumour CXCL12/CXCR4 signalling and immune cell characterisation

In separate experiments, mice received RT/RTCT and concurrent or adjuvant plerixafor but were euthanised immediately at the end of treatment. The tumours were removed to characterise CXCL12/CXCR4 signalling and the immune cell microenvironment. Immunohistochemistry (IHC) was used to evaluate phosphorylated CXCR4 (pCXCR4), phosphorylated AKT (pAKT), phosphorylated ERK (pERK), CD31 (microvessels) and PD-L1 levels. Myeloid cell surface markers were also assessed, including CD11b (immune cells), Ly6G (granulocytic myeloid-derived suppressor cells—MDSCs) and F4/80 (macrophages). The hypoxia marker EF5 (2-(2-Nitro-1H-imidazole-1-yl)-N-(2,2,3,3,3-pentafluoropropyl)acetamide), a gift from Dr C. Koch (University of Pennsylvania), was injected intraperitoneally at a dose of 10 mg/kg 3 hours before euthanising the mice. The IHC antibodies and dilutions are summarised in Supplemental Table 1. Protein expression was reported as the percentage of tumour cell area, stromal area or total area that stained positively in each section, as determined by a small animal pathologist.

Since CXCL12 is a secreted molecule, gene rather than protein expression was assessed using reverse transcription polymerase chain reaction (RT-PCR). Total RNA was extracted from frozen tissue using the Qiagen RNeasy Mini Total RNA Extraction kit (Qiagen, Mississauga). Total RNA was reverse-transcribed using SuperScript III Reverse Transcriptase (Life Technologies). RT-PCR was performed using SYBR Green PCR Master Mix (Life Technologies) with CXCL12 specific primers. The 2-ΔΔ Ct method was used to calculate relative CXCL12 expression, using the L32, HSP90 and YWAZ housekeeping genes as endogenous controls for normalisation. The CXCL12 and housekeeping gene primer sequences are provided in Supplemental Table 2. RT-PCR was run in triplicate for each sample.

### Late intestinal radiation toxicity

To evaluate the general tolerability of high-dose abdominal RT, C57BL/6 non-tumour-bearing mice received whole abdominal RT to a dose of 50 Gy in 2 Gy daily fractions over 5 weeks, using a single 3 × 4 cm treatment field with or without concurrent weekly cisplatin (4 mg/kg) and concurrent plerixafor (5 mg/kg/day). The mice were euthanised 22–29 weeks after completing treatment. The intestines were removed, stained with haematoxylin and eosin (H&E) and examined by a small animal pathologist for histologic evidence of radiation injury.

In a separate series of experiments, C57BL/6 mice were anaesthetised and a small polyethylene catheter (1.6 mm outside diameter, 1 mm inside diameter, SAI Delivery Research Solutions) was inserted 10 mm into the rectum. CT imaging of the catheter was performed to localise the isocenter of a cylindrical radiation treatment volume approximately 5 mm in diameter and 5 mm in length. A single 20 Gy radiation fraction delivered to this volume using a 360° rotational arc. Cisplatin 4 mg/kg was administered as a single ip dose two hours before irradiation. Plerixafor 5 mg/kg/day was administered sc for 30 days beginning 3 days before irradiation. The mice were euthanised 30 days or 90 days after irradiation. The distal colon and rectum were excised, sectioned longitudinally, stained with H&E and examined by a small animal pathologist for histologic evidence of radiation injury using a previously described radiation injury score (RIS), which is summarised in Supplemental Table 3.^11^ The rectum was also examined using IHC for changes in CXCR4 expression and T-cell (CD4, CD8) and myeloid cell (CD11b, Ly6G, F4/80) accumulation.

### Statistical analysis

For the tumour growth delay studies, the times for the individual tumours to regrow to the size at the start of treatment were calculated using linear interpolation between the points closest to the regrowth time, and the median regrowth time was then calculated for each treatment group. The Mann-Whitney test was used to compare tumour regrowth times and IHC expression levels between two treatment groups, and one-way ANOVA with the Newman-Keuls test was used for comparisons among multiple treatment groups. Results with *p* < 0.05 were considered statistically different.

## Results

### Sequencing of RTCT and plerixafor for best primary tumour response

**Fig. 1:**
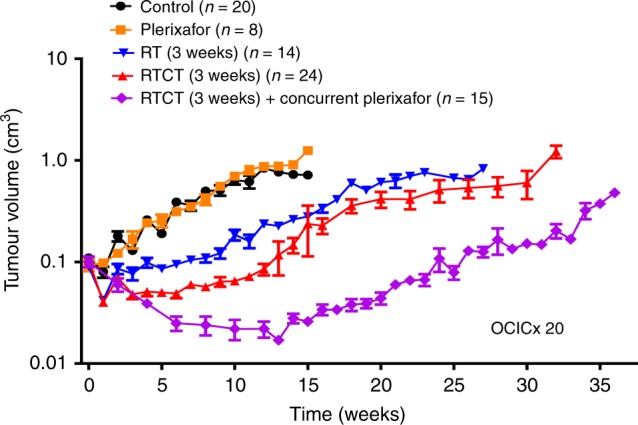
Pooled data from three independent experiments showing OCICx 20 regression and regrowth with RT/RTCT (30 Gy over 3 weeks) and concurrent plerixafor for 3 weeks: no treatment controls (black), plerixafor alone (orange), RT alone (blue), RTCT (red), RTCT and concurrent plerixafor (purple). Each experiment included 5–12 mice per treatment arm. Each data point represents the mean tumour volume in up to 20 mice. A smoothed line was obtained for each treatment group using the ‘loess’ function in R.^34^ Also shown are the mean tumour volume 95% confidence intervals for the RT, RTCT and RTCT + plerixafor arms. Tumour size was monitored after treatment using weekly CT imaging and volume was estimated using orthogonal tumour dimensions, assuming an elliptical shape. Time was measured from the start of treatment. RT, Radiotherapy; RTCT, Radiotherapy and concurrent cisplatin

We previously reported that the combination of RTCT and concurrent plerixafor for 3 weeks was associated with longer tumour growth delay compared to RTCT alone.^4^ We have since further validated this result in separate experiments using the OCICx 20 PDX model. The pooled results of three concurrent OCICx 20 experiments are shown in Fig. 1. RTCT and concurrent plerixafor for 3 weeks substantially enhanced tumour growth delay compared to RTCT alone (median regrowth time 18.8 vs. 13.1 weeks, *p* = 0.02). For perspective, the improvement in tumour response with the addition of concurrent plerixafor to RTCT was greater than the improvement with the addition of concurrent cisplatin to RT alone (the current clinical standard) (median regrowth time 13.1 vs. 9.2 weeks, *p* = 0.04).

**Fig. 2:**
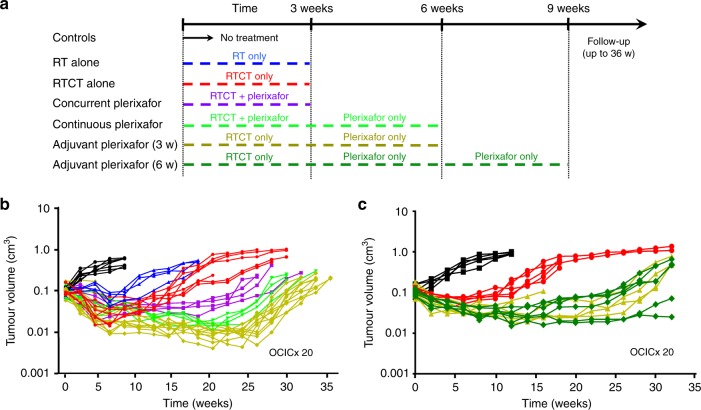
**a** Study schema with RTCT and concurrent, adjuvant or continuous plerixafor. **b** Tumour regression and regrowth of OCICx 20 with RTCT (30 Gy over 3 weeks) and concurrent or adjuvant plerixafor: no treatment controls (black), RT alone (blue), RTCT (red), RTCT and concurrent plerixafor (purple), RTCT and adjuvant plerixafor for 3 weeks beginning immediately after RTCT (yellow), RTCT and continuous plerixafor for 6 weeks (concurrently for 3 weeks and adjuvantly for 3 weeks) (light green). There were 5–12 mice per treatment arm. **2c** Tumour regression and regrowth of OCICx 20 with RTCT (30 Gy over 3 weeks) and 3 vs. 6 weeks of adjuvant plerixafor: no treatment controls (black), RTCT (red), RTCT and adjuvant plerixafor for 3 weeks beginning immediately after RTCT (yellow), RTCT and adjuvant plerixafor for 6 weeks beginning immediately after RTCT (dark green). There were 4–5 mice per treatment arm. In both experiments, tumour size was monitored after treatment using weekly CT imaging and volume was estimated using orthogonal tumour dimensions, assuming an elliptical shape. Time was measured from the start of treatment. 3 w, 3 weeks; 6 w, 6 weeks; 36 w, 36 weeks

The optimal sequencing of RTCT and plerixafor (concurrent, adjuvant or continuous) was evaluated according to the schema outlined in Fig. 2a. In this experiment, RTCT was given for 3 weeks. Plerixafor was given for 3 weeks in the concurrent arm, 3 or 6 weeks in the adjuvant arms and 6 weeks in the continuous arm. All of the RTCT and plerixafor arms showed prolonged tumour growth delay compared to RTCT alone as shown in Fig. 2b, c, with the adjuvant arms showing the greatest improvement (median regrowth time 24.5 weeks for RTCT + concurrent plerixafor, 30.7 weeks for RTCT + adjuvant plerixafor and 15.3 weeks for RTCT alone; *p* = 0.006 and 0.003, respectively). RTCT and adjuvant plerixafor for 3 weeks significantly delayed tumour regrowth compared to RTCT and concurrent plerixafor for 3 weeks (*p* = 0.018). There was a trend towards delayed tumour regrowth with adjuvant plerixafor for 3 weeks compared to continuous plerixafor for 6 weeks (3 weeks during RTCT and 3 weeks after RTCT) (median regrowth time 27.5 weeks), although this did not reach statistical significance (*p* = 0.10). There was no difference in tumour control between 3 and 6 weeks of adjuvant plerixafor (*p* > 0.9).

### Long-term primary tumour control with RTCT and concurrent plerixafor

**Fig. 3:**
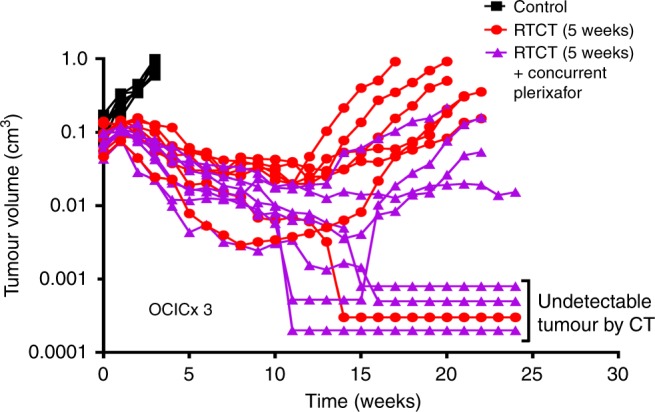
Tumour response of OCICx 3 to dose-intensified RTCT (50 Gy in 25 daily fractions for 5 weeks plus cisplatin 4 mg/kg weekly) with or without concurrent plerixafor (5 mg/kg/day by continuous sc infusion). Tumour size was monitored after treatment using weekly CT imaging and volume was estimated using orthogonal tumour dimensions, assuming an elliptical shape. Imaging findings (including the absence of detectable tumour on CT) were confirmed histologically after the mice were euthanised. Time was measured from the start of treatment. There were 7 mice per treatment group

While tumour growth delay is a relevant and commonly used pre-clinical study endpoint, it does not necessarily correlate with long-term tumour control and cure because of different radiation treatment sensitivities and response kinetics between clonogenic cells (that must be eradicated to achieve cure) and other cell populations. To determine if the combination of RTCT and concurrent plerixafor improved long-term tumour control compared to RTCT alone, we increased the dose of RT (50 Gy in 2 Gy daily fractions over 5 weeks) and concurrent cisplatin (4 mg/kg ip weekly for 5 weeks) and used a different orthotopic cervical cancer PDX model (OCICx 3) to ‘calibrate’ the experiment and ensure that RTCT alone was on the cusp of eradicating the tumours. Long-term control was defined as the absence of tumour re-growth using CT imaging for at least 20 weeks after RTCT and absence of tumour cells in the cervix, uterus or bladder histologically at the time of euthanasia. As shown in Fig. 3, there was a trend towards improved tumour control with the combination of RTCT and plerixafor (3/7 mice, 43%) compared to RTCT alone (1/7 mice, 14%) but this was not statistically significant (p = 0.28).

### End-of-treatment tumour CXCL12/CXCR4 signalling and immune cell characterisation

**Fig. 4:**
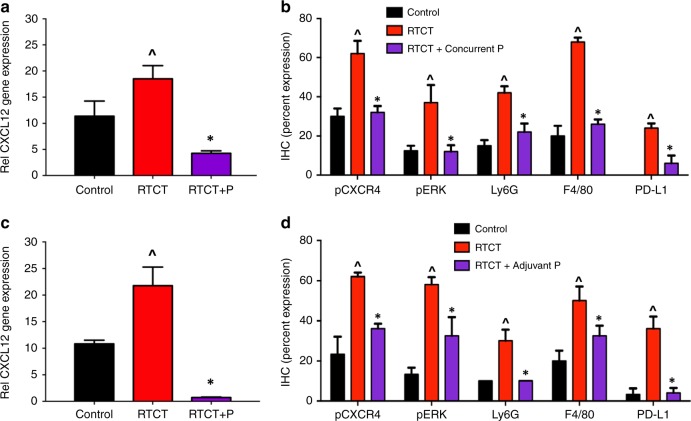
CXCL12 gene expression by RT-PCR at the end of RTCT (30 Gy in 2 Gy daily fractions for 3 weeks plus cisplatin 4 mg/kg weekly) and concurrent (**a**) or adjuvant (**c**) plerixafor (5 mg/kg/day by continuous sc infusion) in OCICx 20. Also shown is protein expression by IHC for pCXCR4 and downstream pERK, markers of MDSC (Ly6G) and macrophage (F4/80) intratumoral accumulation and PD-L1 at the end of concurrent (**b**) and adjuvant (**d**) treatment. The full IHC dataset for the concurrent experiment with additional markers is in Supplemental Fig. 1. Protein expression is shown as the mean ( ± standard error) of the percentage of the tumour cell or stromal surface area that stained positively. pCXCR4, pERK and PD-L1 were predominantly expressed in tumour cell regions, whereas Ly6G and F4/80 were expressed in stroma. The CXCL12 gene expression results are for two tumours per treatment group. The IHC results are for 7–12 tumours per treatment group. RT, radiotherapy; RTCT, radiotherapy and concurrent cisplatin; RTCT + P, RTCT + concurrent or adjuvant plerixafor; ^*p* < 0.05 relative to controls; *p < 0.05 relative to RTCT

To investigate treatment-induced changes in tumour CXCL12/CXCR4 signalling and the immune cell microenvironment, mice bearing the OCICx 20 model were treated with RT or RTCT for 3 weeks (30 Gy) and concurrent or adjuvant plerixafor for 3 weeks. The mice were euthanised at the end of treatment and the primary tumours were excised and analysed using RT-PCR and IHC. The complete dataset for the concurrent plerixafor experiment is shown in Supplemental Fig. 1 and the concurrent and adjuvant results are summarised in Fig. 4. Representative tumour sections showing staining for pCXCR4, Ly6G and F4/80 are shown in Supplemental Fig 2(a), (b) for the concurrent and adjuvant results respectively. In general, tumour cells accounted for 40–60% of the area in each section, with the remainder being tumour stroma. For the IHC studies, pCXCR4, pAKT, pERK and PD-L1 were predominantly expressed in tumour cell regions, whereas CD11b, Ly6G and F4/80 were expressed in stroma. There were no significant changes in the tumour-stroma ratio with RT, RTCT or RTCT and plerixafor.

RTCT alone resulted in increased CXCL12 gene expression. There were significant RTCT-induced increases in pCXCR4, pAKT (assessed in the concurrent experiment only) and pERK protein expression, suggesting CXCR4-dependent activation of downstream cellular pathways. RTCT also increased tumour accumulation of myeloid cell populations, including Ly6G + MDSCs and F4/80 + macrophages, as well as upregulation of PD-L1 expression by tumour cells. There were no changes in the tumour microvasculature (CD31) or tumour hypoxia (EF5) with treatment.

The addition of either concurrent or adjuvant plerixafor abrogated the RTCT-related increases in CXCL12/CXCR4 signalling and myeloid cell accumulation. CXCL12 gene expression, as well as pCXCR4, pAKT, pERK, Ly6G and F4/80 protein expression, returned to near-control levels.

### Late intestinal radiation toxicity

The C57BL/6 mice tolerated whole abdominal RT to a dose of 50 Gy in 2 Gy daily fractions, with or without concurrent weekly cisplatin and concurrent plerixafor, with no change in behaviour or weight (data not shown). There was no histologic evidence of intestinal radiation injury in any of the treatment groups 22–29 weeks after completing treatment (data not shown).

To further investigate sub-acute and late intestinal side effects, a single, targeted RT dose of 20 Gy was administered to the rectum, with or without cisplatin and plerixafor, and the mice were euthanised 30 or 90 days later. A previously described radiation injury score (RIS) based on the seven histologic features listed in Supplemental Table 3 was used to quantitate treatment-induced changes (total score range 0–18).^11^

**Fig. 5:**
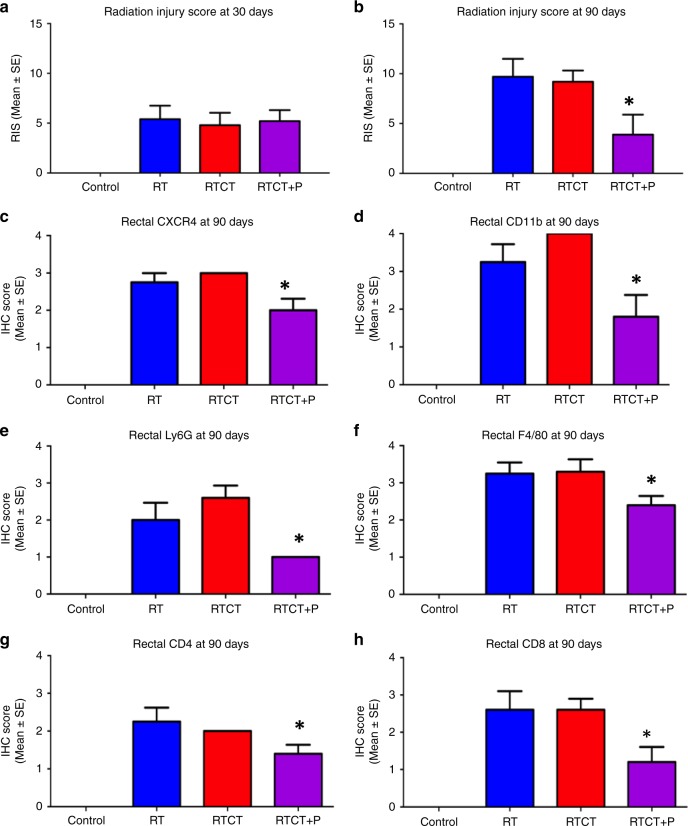
Rectal radiation injury score (RIS) 30 days (**a**) or 90 days (**b**) after RT or RTCT (20 Gy in a single fraction with cisplatin 4 mg/kg ip two hours before) to the rectum of C57BL/6 mice, with or without plerixafor (5 mg/kg/day for 30 days). The 90-days results are pooled from two independent experiments. The results of the two experiments are shown separately in Supplemental Fig. 4. Also shown **c**–**h** is protein expression by IHC for total CXCR4 and markers of immune cell (CD11b), T-cell (CD4, CD8), MDSC (Ly6G) and macrophage (F4/80) infiltration 90 days after RT or RTCT. Protein expression was assessed using a semi-quantitatively scale (range 0–4) that accounted for the number and location (mucosa, submucosa, muscularis) of positively staining cells in the rectum, it is reported as the mean ( ± standard error) score per treatment group. There was no histologic evidence of injury in any of the untreated controls, nor was there measurable CXCR4 or immune cell marker expression. The 30 and 90-day RIS results are for 5 and 10 mice per treatment group respectively. The 90-day IHC results are for 10 mice per treatment group. *p < 0.05 relative to RTCT

Focal histologic changes consistent with rectal mucosal injury were seen in the majority of mice 30 days after RT. The 30-day RIS was zero in the control arm and uniformly higher in the RT, RTCT and RTCT + plerixafor arms with mean values of 5.4, 4.8, 5.2, respectively, as shown in Fig. 5a. The 90-day RISs were measured twice in independent experiments, which yielded similar results. The pooled RISs from the two experiments are shown in Fig. 5b and representative histologic sections of normal colon/rectum illustrating radiation changes at 90 days are shown in Supplemental Fig. 3. The mean 90-day RISs were 0, 9.7 and 9.2 in the control, RT and RTCT arms respectively. However, the RIS was significantly lower in the RTCT + plerixafor arm (mean 3.9, *p* = 0.001 relative to RTCT) indicating radiation protection or mitigation of radiation injury by plerixafor. High (grade 3) levels of rectal vascular sclerosis, wall fibrosis or colitis cystica profunda were seen in 60% of mice in the RT group, 70% of mice in the RTCT group and in none of the mice in the RTCT + plerixafor group.

### End-of-treatment rectal CXCL12/CXCR4 signalling and immune cell characterisation

To investigate the mechanism by which plerixafor prevents or mitigates rectal radiation injury, we evaluated rectal CXCR4 levels and immune cell accumulation 90 days after treatment. This experiment was also done twice, and the pooled results are shown in Fig. 5c–h. In unirradiated control animals, rectal CXCR4 levels were immeasurable and immune cells could not be identified. Similar to what we observed in tumours (Fig. 4), RTCT increased rectal CXCR4 expression and the accumulation of immune cells, including T-cells (CD4, CD8), MDSCs (Ly6G) and macrophages (F4/80). When plerixafor was added to RTCT, there were significant decreases in CXCR4 and in all of the immune cell populations.

## Discussion

The CXCL12/CXCR4 chemokine pathway plays a central role in immune and tumour cell trafficking and has been implicated in tumour development and progression, the development of metastases and RT response.^12^ High pre-treatment tumour CXCL12 or CXCR4 expression is associated with early recurrence and death after potentially curative RT in patients with cervical cancer or other malignancies.^3,12^ In addition, we found that RT dramatically increases tumour CXCL12 expression (Fig. 4) consistent with previous reports,^13–16^ suggesting that pre-treatment CXCL12 levels may not reflect the full biological importance of this pathway. Both CXCL12 and CXCR4 are upregulated by hypoxia,^12^ and RT can cause microvascular changes leading to reduced perfusion and oxygenation.^16,17^ We were unable to detect differences in CD31 or EF5 staining between the control and RT or RTCT arms at the end of treatment to explain the increase in CXCL12, but it is possible that transient, time-dependent changes occurred earlier. However, RT has also been reported to upregulate CXCL12 in a hypoxia-independent manner.^15^ The underlying mechanism is not fully understood but RT-induced DNA damage has recently been reported to directly increase the production of inflammatory cytokines and chemokines through micronucleation at the time of cell division with recruitment of cyclic GMP-AMP synthetase (cGAS) and activation of stimulator of interferon genes (STING).^18^ Interactions between hypoxia and this delayed DNA damage response will be investigated in future experiments to develop biomarkers for future clinical trials and understand when blood and tumour samples should optimally be acquired relative to the start of treatment.

This study builds on our previous results^4^ and provides further evidence that the combination of RTCT and concurrent plerixafor improves tumour response compared to RTCT alone (Figs. 1, 2). We have seen prolonged tumour growth delay in several PDX tumour models with this treatment regimen, including those with either low or high baseline CXCR4 expression,^4^ suggesting that it may be broadly applicable in patients with cervical cancer. Our findings also suggest improved long–term tumour control with CXCR4 inhibition (43% vs. 14%, Fig. 3), implying the potential for higher cure rates in patients. This experiment was underpowered to reliably detect a 29% benefit (>35 mice would have been required per treatment arm) but the results are encouraging and strengthen the rationale for moving this combination into the clinic.

The optimal timing and duration of CXCR4 inhibition relative to RTCT are key considerations in clinical trial design. RTCT and concurrent plerixafor was chosen for our initial experiments^4^ to maximise the likelihood of interaction between the two treatments. Here, we provide the first direct comparison between concurrent plerixafor administered for 3 weeks during fractionated RT/RTCT and adjuvant plerixafor administered for 3 weeks after RT/RTCT. The results (Fig. 2b) indicate improved treatment response regardless of the timing, but a more pronounced effect with adjuvant plerixafor. Interestingly, longer-term adjuvant treatment for 6 weeks was no more effective than shorter-term treatment (Fig. 2c). This implies that CXCR4 inhibitors can be administered after RTCT with the expectation of equivalent if not better tumour control, which has the benefit of minimising concerns about overlapping toxicity. This may be important because CXCR4 inhibitors mobilise hematopoietic stem cells from their normally sequestered and protected location in the bone marrow into circulation,^19^ theoretically making them more vulnerable to cisplatin. To mitigate the (probably very small) risk of late bone marrow aplasia among cervical cancer patients in future clinical trials, plerixafor should be started towards the end of RTCT after the last dose of concurrent cisplatin.

The CXCL12/CXCR4 pathway has wide-ranging effects on intracellular signalling^12^ that could influence RT sensitivity.^20^ However, the results of our timing and duration studies imply that plerixafor is modulating pathways that influence tumour recovery towards the end of and/or early after a course of fractionated RT, rather than intrinsic cellular sensitivity directly. This is consistent with other reports that have shown myeloid cell accumulation during and after RT to strongly influence treatment response.^16,17,21,22^ We identified an increase in Ly6G + MDSCs and F4/80 + macrophages with RT or RTCT alone and a reduction to baseline levels with the addition of either concurrent or adjuvant plerixafor (Fig. 4b, d). Previous reports have demonstrated high levels of CXCR4 expression by these cell populations.^23,24^ MDSCs, macrophages and other myeloid cells may promote cancer recurrence after RT through the production of pro-angiogenic cytokines that enhance the recovery of the tumour vasculature. In pre-clinical brain tumour models, treatment-induced accumulation of CD11b + cells was associated with recovery of tumour perfusion and tumour regrowth after irradiation, which were prevented with the addition of a CXCR4 inhibitor.^16^ A study using the ME180 cervical cancer cell line demonstrated a strong association between high intratumoral MDSC levels, excess production of the pro-angiogenic factor Bv8 and RT resistance; selective depletion of MDSCs significantly enhanced RT response.^25^ While MDSCs also produce local tumour immunosuppression that could contribute to treatment resistance,^26^ this is unlikely to explain the findings because the nude and NRG mice used in these experiments, while having intact chemokine pathways and myeloid cell populations, have impaired adaptive immune systems.^5^ Interestingly, similar results were observed using the TRAMP prostate model in fully immunocompetent C57BL/6 mice,^17^ suggesting that the findings are also likely to apply more broadly in other model systems and in patients with intact immunity.

The rectum and sigmoid are in close proximity to the cervix and concerns about serious intestinal side effects often limit RT dose intensification, even when using high-precision, image-guided treatment techniques. Our group previously reported that plerixafor enhances jejunal crypt cell survival after RT or RTCT, in keeping with a reduction in acute intestinal toxicity.^4^ Here, we demonstrate that plerixafor also reduces histologic changes in the rectum (Fig. 5 and Supplemental Fig. 3) that are commonly associated with late RT toxicity.^11^ We identified evidence of rectal radiation injury 30 days after a single RT dose of 20 Gy, a higher level of injury at 90 days and a protective effect of plerixafor at 90 days. Further study is needed to better define the dose-response and time course of plerixafor-related intestinal protection. Interestingly, others have shown radioprotection of normal skin, lung and brain with CXCL12/CXCR4 inhibition,^27–29^ suggesting that this may be a broadly translatable strategy to limit RT side effects. The mechanisms responsible for this protective effect have not been established but may relate to reversal of the sustained normal tissue inflammatory response caused by radiation. We identified upregulation of the CXCL12 pathway with accumulation of CXCR4-expressing inflammatory cells following RT, and reversal of these changes with plerixafor (Fig. 5). Furthermore, mesenchymal stem cells (MSCs), which similarly express CXCR4 at high levels,^30^ are known to accumulate in inflamed intestine and mitigate long-term injury.^31^ The relative balance between the pro-inflammatory immune cell response, including macrophage polarisation towards the M2 state,^32^ and the anti-inflammatory MSC response will be investigated in future experiments.

## Conclusion

In conclusion, this study demonstrates that RTCT increases CXCL12/CXCR4 signalling in cervical cancer, leading to intratumoral accumulation of MDSCs and other myeloid cell populations that may contribute to disease recurrence. Furthermore, it provides the strongest possible evidence using clinically relevant tumour models and treatment protocols^33^ that inhibiting CXCL12/CXCR4 signalling during or immediately after RTCT improves treatment response and possibly long-term disease control without increasing (and perhaps even reducing) side effects. The results provide a strong rationale for future clinical trials of RTCT and plerixafor or another CXCL12/CXCR4 inhibitor aimed at improving the efficacy of frontline curative therapy for patients with cervical cancer and possibly other malignancies.

## Supplementary information

Lecavalier and Chaudary, Cervix Plerixafor Supplemental Material

## Data Availability

Data will be made available upon request.
