# Correction: Targeting CXCL12/CXCR4 and myeloid cells to improve the therapeutic ratio in patient-derived cervical cancer models treated with radio-chemotherapy

**DOI:** 10.1038/s41416-019-0545-z

**Published:** 2019-08-09

**Authors:** Magali Lecavalier-Barsoum, Naz Chaudary, Kathy Han, Melania Pintilie, Richard P. Hill, Michael Milosevic

**Affiliations:** 1 Segal Cancer Centre, Jewish General Hospital and McGill University, Montreal, Canada; 2 University Health Network and Princess Margaret Cancer Centre, Toronto, Canada; 3 Department of Radiation Oncology, University of Toronto, Toronto, Canada; 4 Institute of Medical Science, University of Toronto, Toronto, Canada; 5 Dalla Lana School of Public Health, University of Toronto, Toronto, Canada; 6 Department of Medical Biophysics, University of Toronto, Toronto, Canada

**Keywords:** Radiotherapy, Cancer microenvironment, Tumour immunology

**Correction to**: *British Journal of Cancer* (2019) **121**, 249–256; 10.1038/s41416-019-0497-3; www.bjcancer.com; published online 26 June 2019

Since the publication of this paper, the authors have reported that an incorrect version of Fig. 1 was presented. The correct version of Fig. 1 is provided below.

**Fig. 1:**
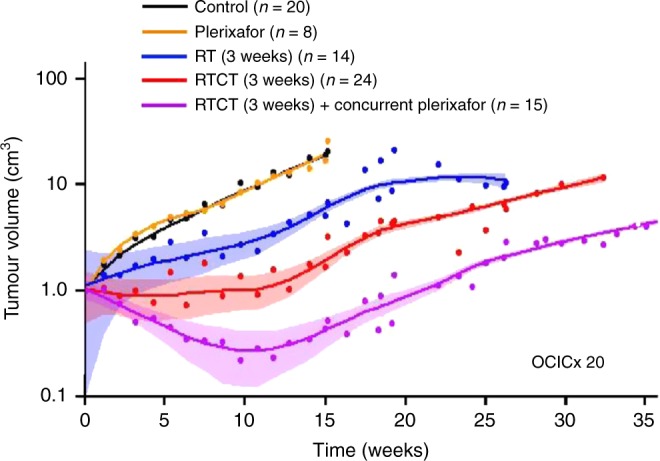
Pooled data from three independent experiments showing OCICx 20 regression and regrowth with RT/RTCT (30 Gy over 3 weeks) and concurrent plerixafor for 3 weeks: no treatment controls (black), plerixafor alone (orange), RT alone (blue), RTCT (red), RTCT and concurrent plerixafor (purple). Each experiment included 5–12 mice per treatment arm. Each data point represents the mean tumour volume in up to 20 mice. A smoothed line was obtained for each treatment group using the ‘loess’ function in R.^34^ Also shown are the mean tumour volume 95% confidence intervals for the RT, RTCT and RTCT + plerixafor arms. Tumour size was monitored after treatment using weekly CT imaging and volume was estimated using orthogonal tumour dimensions, assuming an elliptical shape. Time was measured from the start of treatment. RT: Radiotherapy; RTCT: Radiotherapy and concurrent cisplatin

