# Supplementary material for: Targeting CXCL12/CXCR4 and myeloid cells to improve the therapeutic ratio in patient-derived cervical cancer models treated with radio-chemotherapy
Source: Br J Cancer. 2019 Jun 26;121(3):249–56. doi: 10.1038/s41416-019-0497-3 (PMC6738100; doi:10.1038/s41416-019-0497-3)
Supplement: Supplementary file 1 — Lecavalier and Chaudary, Cervix Plerixafor Supplemental Material [file 41416_2019_497_MOESM1_ESM.pdf]

Supplemental Table 1: Immunohistochemistry (IHC) antibodies and dilutions

IHC Marker	Antibody	Dilution
CXCR4	Santa Cruz<br>Goat anti-human	1:400
pCXCR4	Abcam<br>Rabbit anti-human	1:300
Ly6G	Invitrogen<br>Rat anti-human	1:200
PD-L1	Roche<br>Rabbit anti-human	1:200
pERK	Cell Signalling<br>Rabbit anti-human	1:50
pAKT	Cell Signalling<br>Rabbit anti-human	1:50
EF5	Cameron Koch, University of Pennsylvania<br>Mouse anti-human	1:50
F4/80	Bio-Rad<br>Rat anti-mouse	1:2000
CD31	Santa Cruz<br>Rabbit anti-human	1:1000
CD11b	Abcam<br>Rabbit anti-human	1:100
CD4	Ebioscience<br>Mouse anti-human	1:200
CD8	Abcam<br>Rabbit anti-mouse	1:1000

Supplemental Table 2: CXCL12 and housekeeping gene primer sequences for the RT-PCR gene expression studies

Primers	Forward Sequence (5`-3`)	Reverse Sequence (3`-5`)
CXCL12	TGCATCAGTGACGGTAAACCA	CACAGTTTTGGAGTGTTGAGGAT
L32	GCCATTGTAGAAAGAGCAGCA	TGCACACAAGCCATCTACTCA
YWAZ	CTGCCTACATATTGGTGTGTG	TTTGTGTCACAGCCTCACAAG
HSP90	GAGGCAGACAAAAACGACAAA	TGAGAAACCAGAGGAGAGCAG

Supplemental Table 3: Radiation Injury Score (RIS) adapted from Langberg *et al.* 1992

Histologic Feature	Range
Epithelial atypia	0-3
Mucosal ulceration	0-2
Serosa thickening	0-3
Vascular sclerosis	0-3
Lymphatic congestion	0-1
Rectal wall fibrosis	0-3
Colitis cystica profunda	0-3
**Total Radiation Injury Score (RIS)**	**0-18**

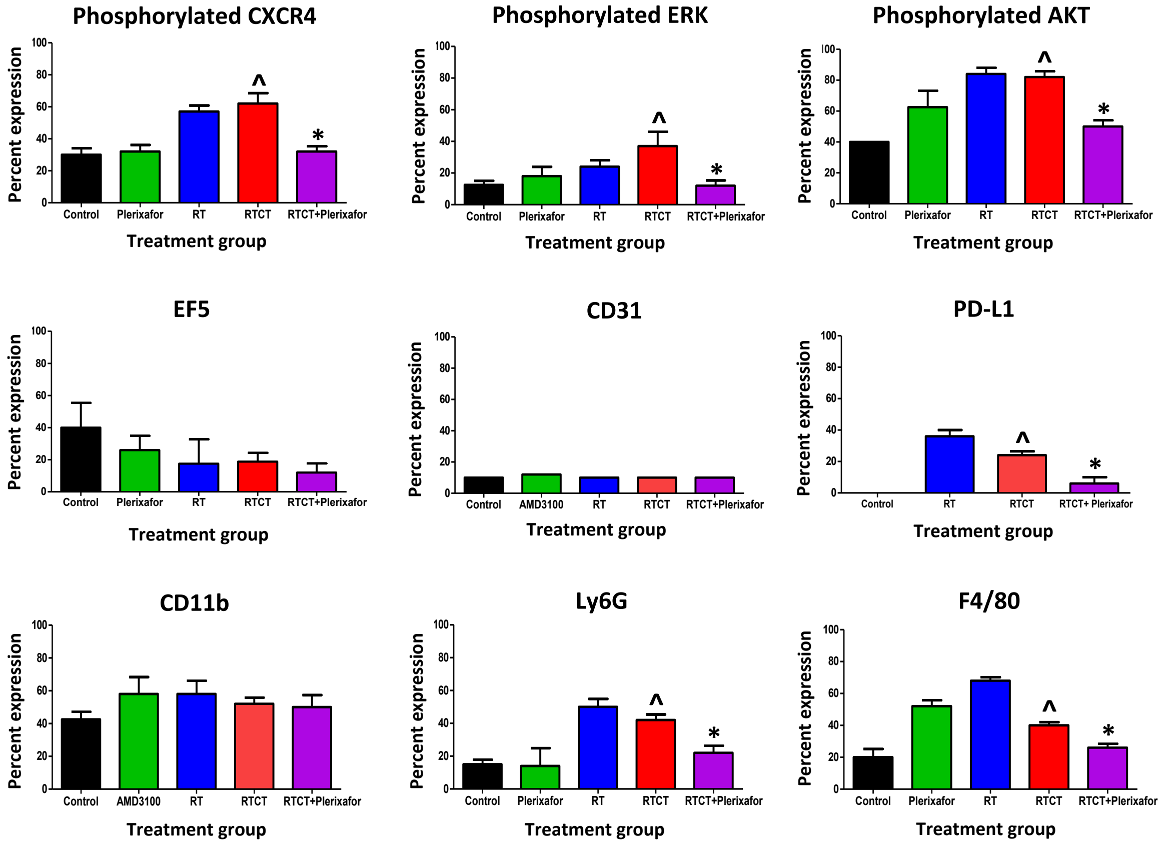

Supplemental Figure 1: Protein expression by IHC for pCXCR4, pAKT, pERK, vascular density (CD31), hypoxia (EF5), markers of myeloid cell (CD11b), MDSC (Ly6G) and macrophage (F4/80) intratumoral accumulation and PD-L1 at the end of RTCT (30 Gy in 2 Gy daily fractions for 3 weeks plus cisplatin 4 mg/kg weekly) and concurrent Plerixafor (5 mg/kg/day by continuous sc infusion). Protein expression is shown as the mean (± standard error) of the percentage of the tumor cell or stromal surface area that stained positively. pCXCR4, pAKT, pERK and PD-L1 were predominantly expressed in tumor cell regions, whereas CD11b, Ly6G and F4/80 were expressed in stroma. The results are for 7-12 tumors per treatment group. RT: Radiotherapy; RTCT: Radiotherapy and concurrent cisplatin; RTCT+P: RTCT + concurrent Plerixafor; ^ $p<0.05$ relative to controls; * $p<0.05$ relative to RTCT

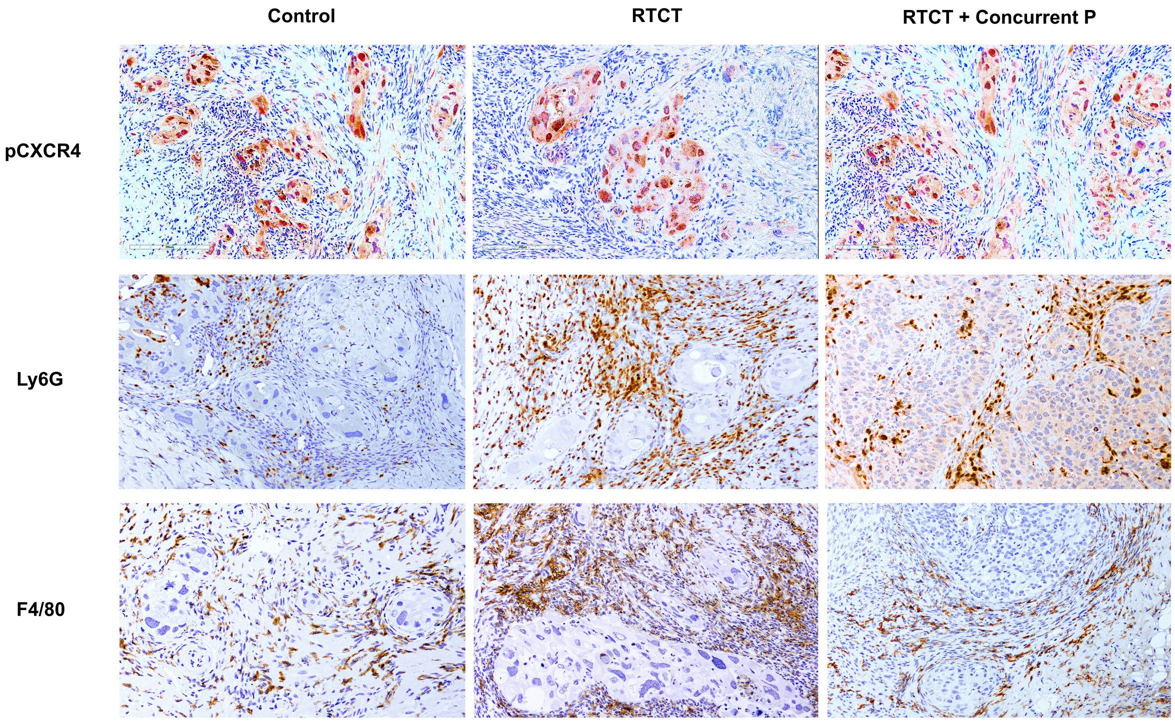

Supplemental Figure 2(a) – RTCT and Concurrent Plerixafor: Representative tumor sections showing immunohistochemical (IHC) staining for phosphorylated CXCR4 (pCXCR4), Ly6G+ MDSCs and F4/80+ macrophages in the control (no treatment), RTCT, and RTCT + plerixafor treatments arms. Mice in the control arm were euthanized when the tumors reached a size of 1-1.5 cm. Mice in the RTCT and RTCT + concurrent plerixafor arms were euthanized immediately after completing all treatment (3 weeks). Magnification 20X.

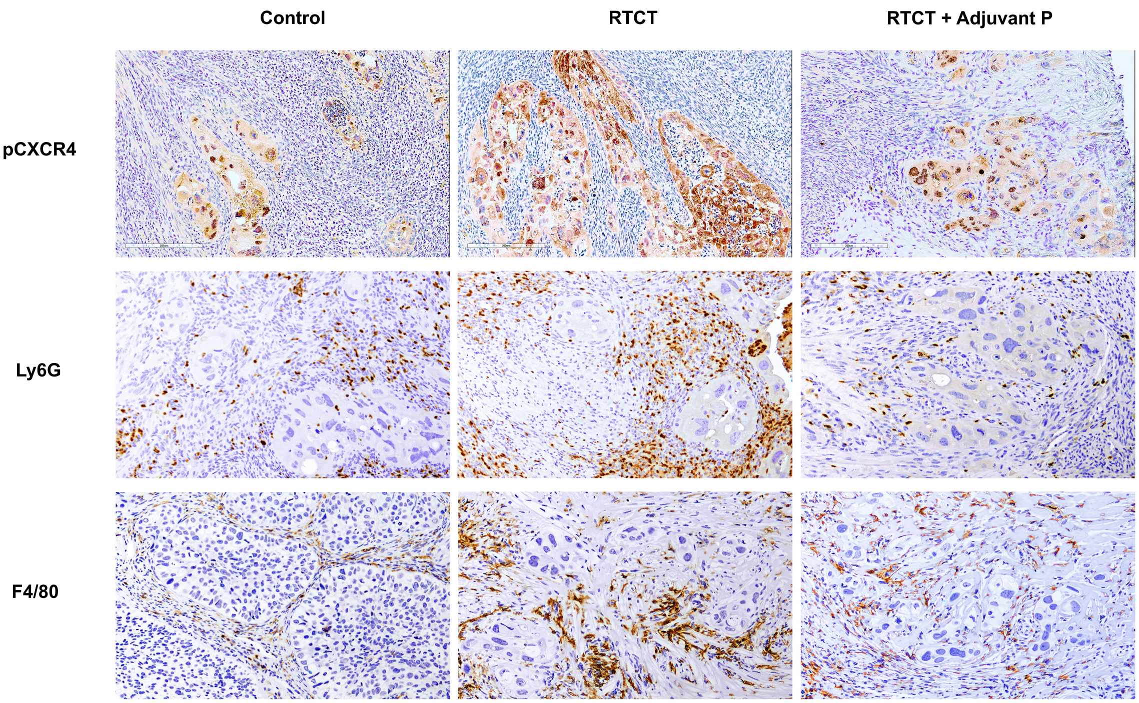

Supplemental Figure 2(b) – RTCT and Adjuvant Plerixafor: Representative tumor sections showing immunohistochemical (IHC) staining for phosphorylated CXCR4 (pCXCR4), Ly6G+ MDSCs and F4/80+ macrophages in the control (no treatment), RTCT, and RTCT + plerixafor treatments arms. Mice in the control arm were euthanized when the tumors reached a size of 1-1.5 cm. Mice in the RTCT arm were euthanized immediately after completing all treatment (3 weeks). Mice in the RTCT + adjuvant plerixafor arm were euthanized immediately after completing plerixafor (6 weeks). Magnification 20X.

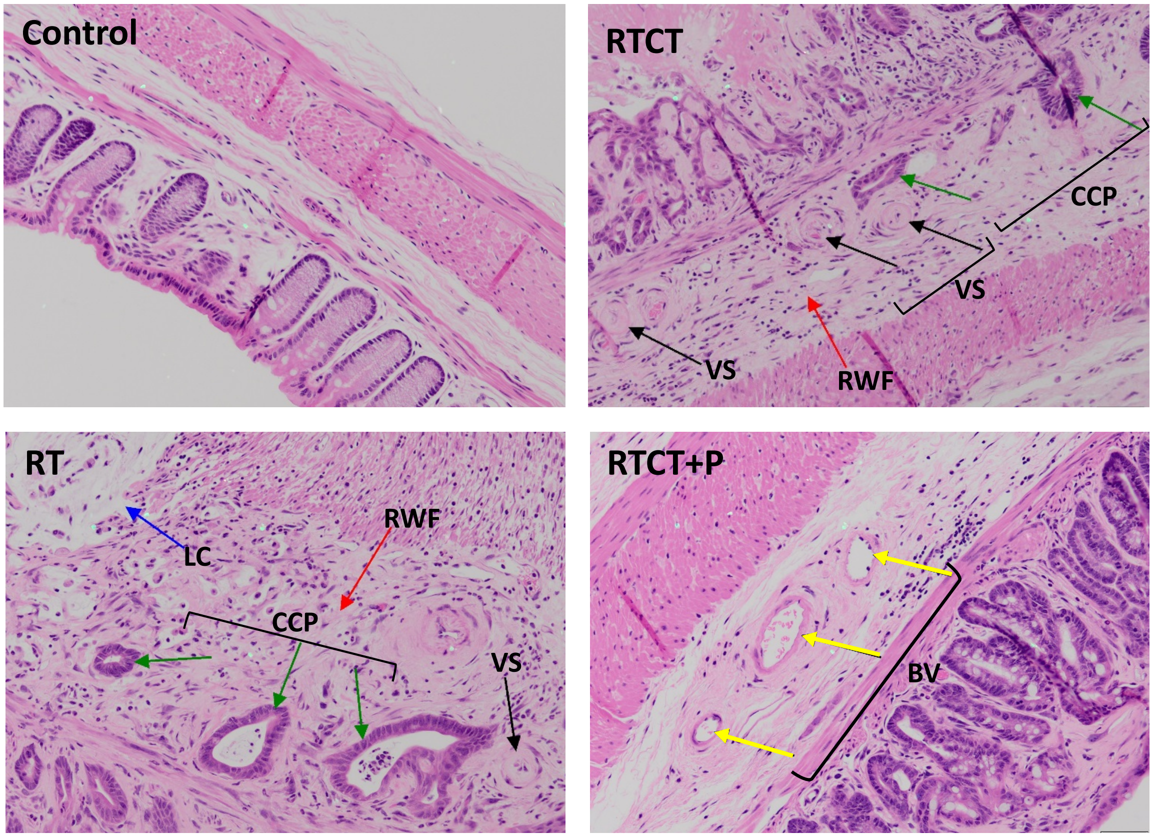

Supplemental Figure 3: Representative sections of normal mouse colon/rectum showing histologic changes consistent with late radiation injury (Supplemental Table 3, Langberg *et al.* 1992) 90 days after RT, RTCT or RTCT + plerixafor. Green arrows: Colitis cystica profunda (CCP); Blue arrows: Lymphatic congestion (LC); Black arrows: Vascular sclerosis (VS); Red arrows: Rectal wall fibrosis (RWF); Yellow arrows: Histologically unremarkable (unaffected) blood vessels (BV). RT was administered as a single 20 Gy fraction. Cisplatin 4 mg/kg was administered as a single ip dose two hours before irradiation. Plerixafor 5 mg/kg/day was administered sc for 30 days beginning 3 days before irradiation. Magnification 20X.
